# Glycoengineering artificial receptors for microglia to phagocytose Aβ aggregates†

**DOI:** 10.1039/d0sc07067j

**Published:** 2021-02-23

**Authors:** Dongqin Yu, Chun Liu, Haochen Zhang, Jinsong Ren, Xiaogang Qu

**Affiliations:** Laboratory of Chemical Biology, State Key Laboratory of Rare Earth Resource Utilization, Changchun Institute of Applied Chemistry, Chinese Academy of Sciences Changchun Jilin 130022 P. R. China xqu@ciac.ac.cn; University of Science and Technology of China Hefei Anhui 230029 P. R. China

## Abstract

Oligomeric and fibrillar amyloid-β (Aβ) are principally internalized *via* receptor-mediated endocytosis (RME) by microglia, the main scavenger of Aβ in the brain. Nevertheless, the inflammatory cascade will be evoked after vast Aβ aggregate binding to pattern recognition receptors on the cell membrane, which then significantly decreases the expression of these receptors and further deteriorate Aβ deposition. This vicious circle will weaken the ability of microglia for Aβ elimination. Herein, a combination of metabolic glycoengineering and self-triggered click chemistry is utilized to engineer microglial membranes with ThS as artificial Aβ receptors to promote microglia to phagocytose Aβ aggregates. Additionally, to circumvent the undesirable immune response during the process of the bioorthogonal chemistry reaction and Aβ-microglial interaction, Mn-porphyrin metal–organic frameworks (Mn-MOFs) with superoxide dismutase (SOD) and catalase (CAT) mimic activity are employed to carry *N*-azidoacetylmannosamine (AcManNAz) and eradicate over-expressed reactive oxygen species (ROSs). The artificial Aβ receptors independent of a signal pathway involved in immunomodulation as well as Mn-MOFs with antioxidant properties can synergistically promote the phagocytosis and clearance of Aβ with significantly enhanced activity and negligible adverse effects. The present study will not only provide valuable insight into the rational design of the microglial surface engineering strategy *via* bioorthogonal chemistry, but also hold great potential for other disease intervention associated with receptor starvation.

## Abstract 2

A combination of metabolic glycoengineering and self-triggered click chemistry is utilized to engineer a microglial membrane with ThS as artificial Aβ receptors to promote microglia to phagocytose Aβ aggregates.

## Graphical abstract

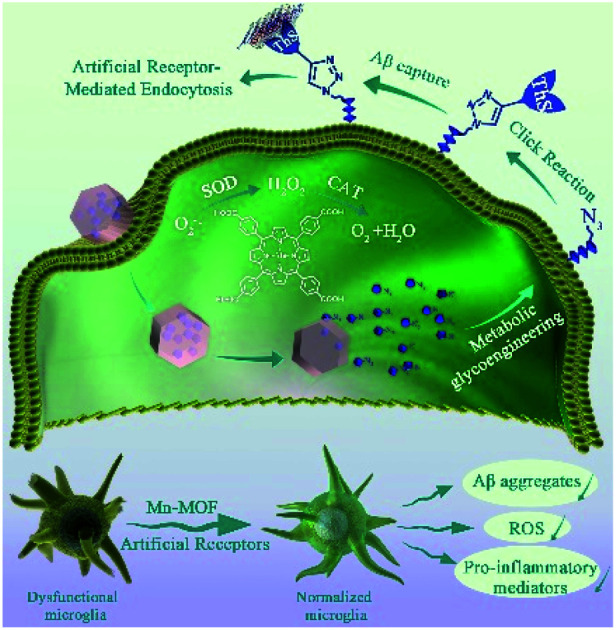

## Introduction

Amyloid β (Aβ) peptides, which are produced from amyloid precursor protein (APP) cleaved by β- and γ-secretase can be metabolized from the brain to maintain their dynamic equilibrium.^1^ The abnormal accumulation of extracellular amyloid β (Aβ) is considered to be one of the critical pathological hallmarks of Alzheimer's disease (AD).^2,3^ As the endocranial resident macrophages, microglia constantly survey the brain microenvironment for pathogens and play essential roles in phagocytosis and degradation of intracranial Aβ.^4^ Microglia express Aβ-related cell surface receptors such as scavenger receptors (SRs), toll-like receptors (TLRs), formyl peptide receptors (FPRs), receptors for advanced glycation end products (RAGE), lipoprotein receptor-related proteins (LRPs) and triggering receptors expressed on myeloid cells 2 (TREM-2) for Aβ phagocytosis, especially oligomeric and fibrillar Aβ.^5^ Microglial clustering found in the brain before the symptoms of AD manifest delays AD progression by accelerating Aβ clearance.^6^ However, with AD progressing, vast misfolded Aβ binds to pattern recognition receptors on the microglial membranes and then evokes an innate immune response, which in turn significantly decrease the expression of Aβ receptors and increase the production of ROS and pro-inflammatory mediators.^7,8^ Such a negative feedback loop between microglial dysfunction and Aβ clearance will cause the imbalance between Aβ anabolism and catabolism, and finally Aβ deposition. Hence, facilitating Aβ clearance utilizing microglia and normalizing the priming of microglia simultaneously contribute to recovering proteostasis equilibrium, but still remain a great challenge. The existing microglia-based therapeutic strategies have been focused on active or passive immune approaches, epigenetics technologies, or genetic engineering.^9–11^ Regardless of the tremendous progress, these treatments can be technically difficult and laborious with unpredictable outcomes such as potential off-target effects, high cost and low effectiveness, disturbance of the normal signaling pathway and inscrutable immune response. Alternatively, nanotechnology-based approaches can conquer the aforementioned weaknesses and show huge advantages for promoting microglia to clear up Aβ.^12–15^ However, this method relies on passive endocytosis of nanoparticles by microglia after Aβ is captured, which showed limited therapeutic effect. Thus, we speculate that the construction of artificial receptors on the microglial membrane may provide an alternative method to promote Aβ phagocytosis by microglia.^16,17^

Recently, biorthogonal click reaction has attracted much attention owing to its high efficiency, fast kinetics, mild reaction conditions and orthogonality in chemical biology and pharmaceutical science.^18^ Biorthogonal click reaction has been applied in living systems for bioconjugation,^19^ biosensing,^20^ drug synthesis^21^ and other biological processes manipulating.^22^ Particularly, along with metabolic glycoengineering, glycan imaging and glycoproteomic profiling can be realized for disease diagnosis and therapeutics by immobilizing azido-sugars to the cell membrane on the basis of their intrinsic metabolic path.^23–26^ Furthermore, metabolic glycan labelling and biorthogonal click reaction have also been applied to regulate cell function.^27–29^ For example, our group has reported reversible regulation of cell–cell interactions *via* photo-responsive host–guest recognition after engineering cell membranes with β-cyclodextrin and azobenzene.^27^ In addition, Xing *et al.* have reported to manipulate membrane channel activity after engineering cell membranes with lanthanide-doped upconversion nanocrystals.^28^ Metabolic glycoengineering of un-natural sugars together with biorthogonal click reaction also provides a powerful tool to artificially introduce chemical receptors onto the cell surface.^30^

**Scheme 1:**
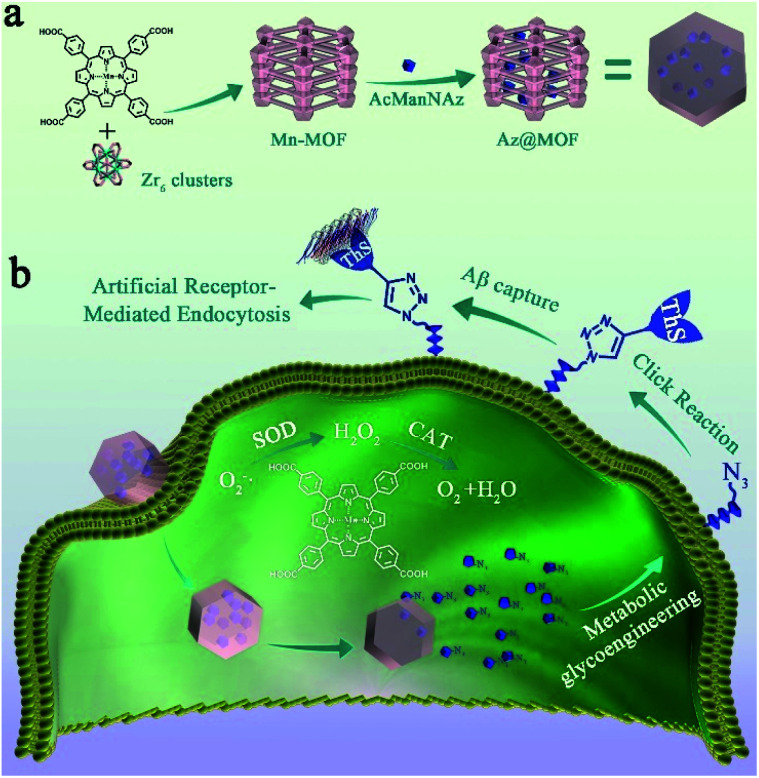
(a) Synthetic process and composition of AcManNAz@Mn-MOFs (Az@MOF); (b) clearance mechanism of Aβ aggregates. First, Az@MOF was endocytosed in microglia and AcManNAz was released. Next, metabolic glycoengineering and biorthogonal click reaction were applied to modify ThS on the microglial membrane. Then, artificial Aβ-receptors ThS could capture Aβ aggregates just like natural Aβ-receptors on the microglial membrane. Simultaneously, SOD and CAT mimic Mn-MOFs could eradicate O_2_˙^−^ and H_2_O_2_ to decrease the levels of ROSs and pro-inflammatory mediators during the process of Aβ capture and degradation.

Herein, a novel non-genetic cell-surface engineering method was presented to promote Aβ clearance using the metabolic glycan labeling technique and click reaction. Specifically, *N*-acetylmannosamine analogues bearing an azide (*N*-azidoacetylmannosamine, AcManNAz) were encapsulated in Zr-based metal–organic frameworks linked by manganese porphyrin (Mn-MOFs) and delivered into microglia for being metabolically incorporated into cell-surface glycans. Then, an alkyne-modified ThS, a thioflavine dye that can selectively capture Aβ aggregates,^37^ was conjugated with AcManNAz *via* self-triggered biorthogonal copper(i)-catalysed azide–alkyne cycloaddition (CuAAC). Notably, the typical concentration of Cu is 0.4 mM in Aβ plaques, which is enough for catalyzing CuAAC.^31^ As artificial Aβ-receptors on the microglial membrane, ThS could capture extracellular Aβ aggregates and improve the ability of microglia to phagocytose Aβ through receptor-mediated endocytosis (RME). To circumvent the over-activation of microglia during the process of the click reaction and Aβ phagocytosis, the manganese porphyrin linkers in the Mn-MOF carrier would mimic the function of natural SOD and CAT to shift microglial phenotypic transition to the neuroprotective subtype through regulating redox equilibrium (Scheme 1). Antioxidative Mn-MOFs could avoid microglial over-activation and maintain the normal functions of microglia during the process of Aβ clearance. With the synergy between bioorthogonal chemistry-engineered artificial Aβ receptors and SOD and CAT mimic Mn-MOFs, microglial capability to eliminate Aβ can be regained and accelerated without over-activated adverse effects.

## Results and discussion

**Fig. 1:**
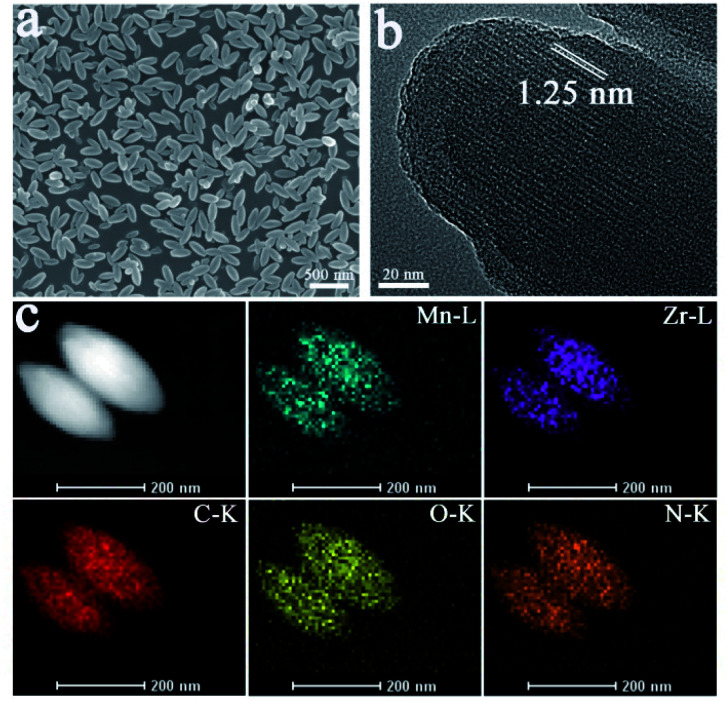
(a) Scanning electron microscopy (SEM) image of Mn-MOFs; (b) high-resolution transmission electron microscopy (TEM) image of Mn-MOFs. The channel size of the Mn-MOFs is highlighted in white; and (c) TEM elemental mapping of C K edge, N K edge, O K edge, Mn L edge, and Zr L edge signals in the synthesized Mn-MOFs.

Prior to the synthesis of Mn-MOFs, Mn-metallized tetrakis(4-carboxyohenyl)porphyrin (Mn-TCPP) was synthesized as previously described (Fig. S1†).^32^ Fourier transformation infrared spectroscopy (FT-IR), mass spectroscopy (MS) and ^1^H NMR demonstrated the successful preparation of Mn-TCPP (Fig. S2–S5†). Then Mn-MOFs were synthesized according to the synthetic process of PCN-224 with Mn-TCPP instead of TCPP.^33^ The powder X-ray diffraction (PXRD) pattern of Mn-MOFs was consistent with the simulated structure of PCN-223 (Fig. S6†).^34^ Scanning electron microscopy (SEM) imaging indicated the extremely homogeneous morphology with a length of 200 nm and a width of 75 nm (Fig. 1a). Furthermore, transmission electron microscopy (TEM) images of Mn-MOFs in Fig. 1b directly displayed a pore size of 1.25 nm, which is in accordance with the result of N_2_ adsorption–desorption (1.25 nm) in Fig. S7.† The pore size of 1.25 nm permitted the encapsulation and release of AcManNAz (MW = 430). The elemental mapping analysis demonstrated the distribution of Mn and Zr in MOFs (Fig. 1c). X-ray photoelectron spectroscopy (XPS) further presented the existence of the Mn element (Fig. S8†). Furthermore, there was an intense peak (S_0_–S_2_ absorption process) at 466 nm and two *Q*-bands (S_0_–S_1_) after Mn ion chelating, different from free TCPP with an intense Soret band at 417 nm and at 520 nm, 556 nm, 593 nm and 650 nm (Fig. S9†). In addition, the fluorescence emission of Mn-MOFs was negligible (Fig. S10†). According to the previous reports, manganese porphyrin derivatives originating from natural SOD show inherent SOD mimetic activity and CAT mimetic activity.^35,36^ To validate the SOD and CAT activity of Mn-TCPP after being integrated into MOFs, a modified NBT assay was conducted. As shown in Fig. S11,† both Mn-TCPP and Mn-MOFs showed obvious SOD activity. Also, the SOD activity displayed favourable concentration-dependent properties. Furthermore, Mn-MOFs possessed stronger ability to scavenge O_2_˙^−^ at the same concentration compared with free Mn-TCPP, which was attributed to their well-isolated Mn-TCPP molecules and porous structure. As for their CAT activity, the same results were obtained (Fig. S12†).

To encapsulate AcManNAz in Mn-MOFs, the resulting Mn-MOFs were mixed with AcManNAz in ethanol solution to obtain AcManNAz@Mn-MOFs (Az@MOF). Mn-MOFs and AcManNAz with different ratios were mixed and the amount of encapsulated AcManNAz was determined using high-performance liquid chromatography (HPLC) (Table S1†). The amount of encapsulated AcManNAz increased with the increase of the ratio of AcManNAz to Mn-MOFs, and the encapsulated AcManNAz approached a saturation point at 0.85 μmol mg^−1^ Mn-MOFs when the ratio of AcManNAz to Mn-MOFs reached 9.3 μmol mg^−1^. Hence, the maximal encapsulation amount of 0.85 μmol mg^−1^ was selected for the following experiments. Also, the SEM imaging showed well-maintained morphology of Mn-MOFs after encapsulating AcManNAz (Fig. S13†). The crystallinity and structural integrity of Mn-MOFs were well-maintained after AcManNAz was encapsulated in Mn-MOFs (Fig. S6†). Thus, the well-isolated ligands and inherent porous periodic arrays not only were conducive to a better nanozyme activity but also provided the cavity for encapsulating AcManNAz.

Our previous study has demonstrated that neurotoxic copper accumulated in Aβ plaques could effectively catalyze an azide–alkyne biorthogonal cycloaddition reaction when Cu-Aβ aggregates were used as the catalysts.^31^ Indeed, the fluorescence intensity of the click reaction increased about 20-fold after 10 minutes of reaction (Fig. S14†).

To confirm the internalization of Mn-MOFs by microglia, RhB@MOF was first synthesized with rhodamine B (RhB) instead of AcManNAz. Initially, encapsulation of RhB into Mn-MOFs largely suppressed the emission of RhB probably due to the “self-quenching” effect. After RhB@MOF was phagocytosed and RhB was efficiently released, the emission of RhB could be restored. Fig. S15 and S16† manifest that Mn-MOFs could enter the BV2 microglial cell and release RhB in a time-dependent manner. Through further quantitative analysis of the release profile according to the result of the flow cytometry assay (Fig. S17†), RhB could be continuously released after RhB@MOFs were endocytosed by microglia during 48 hours of incubation time. The persistent drug release was the consequence of the porosity of Mn-MOFs. Although there was no stimulant to trigger boosting release in the cells, the diffusion of RhB through the pores of Mn-MOFs rather than explosive responsive release helps in continuous intracellular drug replenishment. MTT assay revealed the excellent biocompatibility of Mn-MOFs as more than 80% of BV2 cells maintained vitality at a concentration of 60 μg mL^−1^ (Fig. S18†).

**Fig. 2:**
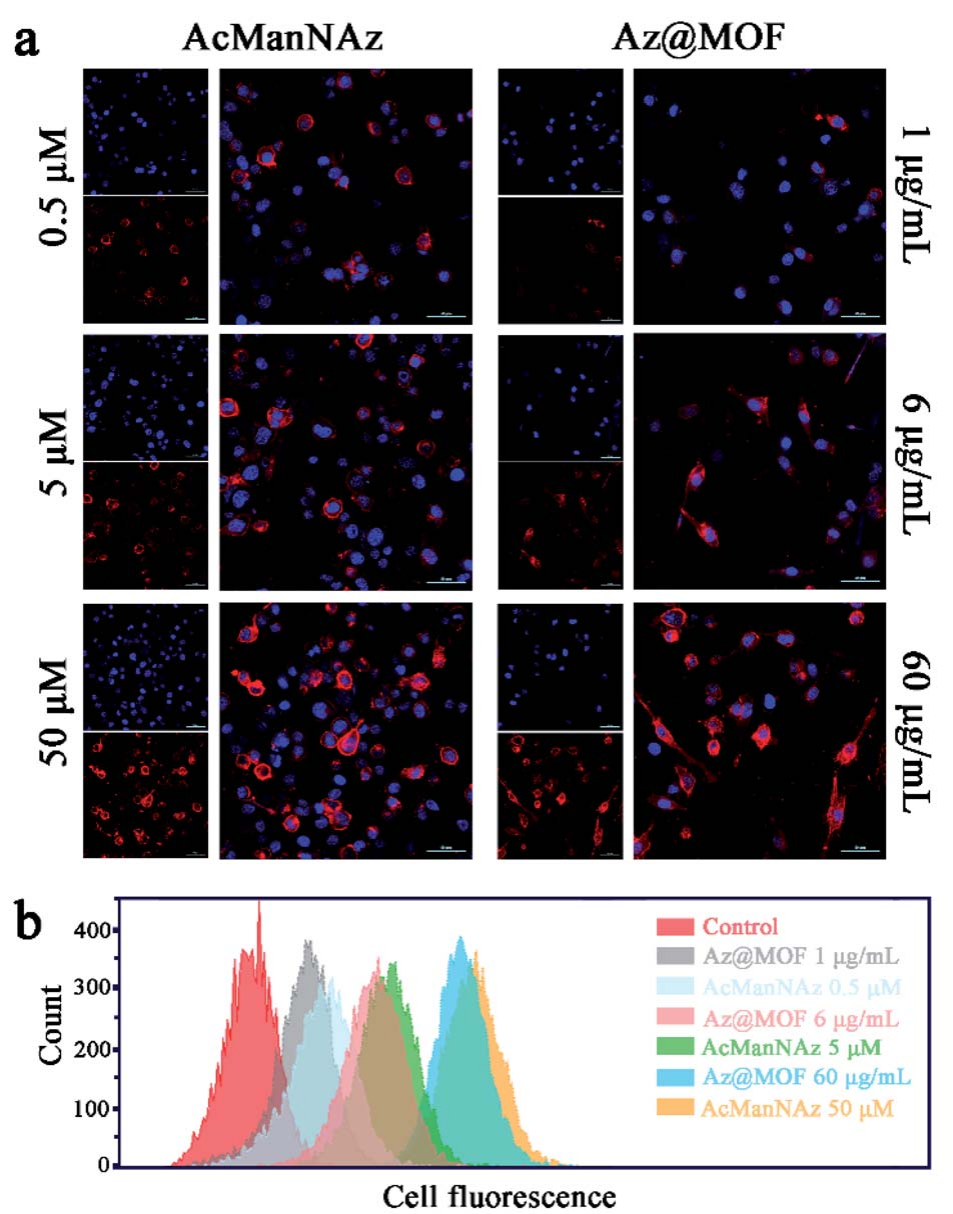
Characterization of azido groups on the microglial membrane with alk-Cy5 as the probe. (a) CLSM imaging of azido groups on the microglial membrane with different concentrations of AcManNAz or Az@MOF (scale bar: 50 μm). (b) Flow cytometry assay of the amount of the azido groups on the microglial membrane with different concentrations of AcManNAz or Az@MOF.

As a prerequisite, we investigated whether the metabolic substrate AcManNAz could be incorporated into mucin-type O-linked glycol-proteins in the microglial membrane through a metabolic glycan labeling technique with an alkynyl-based probe, alk-Cy5. Typically, BV2 cells were treated with AcManNAz or Az@MOF for three days. The concentration of AcManNAz in Az@MOF was kept the same as that of free AcManNAz. Then, the alk-Cy5 probe was added to determine the presence of azide groups on the microglial surface by confocal laser scanning microscopy (CLSM) imaging. The Cy5 fluorescence intensity reflected the amount of azide groups on the microglial surface. As seen from Fig. 2a, the fluorescence signal intensity from Cy5 increased with the increase of the dose of treated AcManNAz and Az@MOF when the amount of alk-Cy5 was kept the same and excess. The same results were obtained from the flow cytometry analysis (Fig. 2b). These results suggested that the amount of azide groups on the microglial surface could be adjustable in a dose-dependent manner. In consideration of the results of the MTT assay, in order to maximize the amount of ThS on the cell surface and minimize cytotoxicity of Mn-MOFs as possible, we chose 60 μg mL^−1^ of Mn-MOFs (containing *ca.* 51 μM of AcManNAz) to carry out the next experiments.

According to previous studies, the half-life of AcManNAz on the cell surface was about 17.4 h.^27^ Therefore, carriers that can realize sustained drug release are beneficial for continuous AcManNAz replenishment on the cell membrane to extend its retention time. To further evaluate the attenuation of the AcManNAz on the cell membrane, we conducted flow cytometry to quantify the amount of AcManNAz on the microglial membrane. AcManNAz or Az@MOF was first added to co-incubate with BV2 cells for 3 days, respectively, and then, the old medium was replaced with fresh medium without AcManNAz or Az@MOF. We labelled the remaining surface-associated AcManNAz with alk-Cy5 over 4 days. As shown in Fig. S19 and S20,† the fluorescence intensity of Cy5 decreased more slowly with the increase of incubation time in the Az@MOF group than the AcManNAz group. For the Az@MOF group, AcManNAz on the cell membrane could be maintained at 20% on the fourth day because of the ability of sustained drug release of Mn-MOF. However, it could only be maintained at 1.8% in the AcManNAz group. Thus, the unusual slow-release behaviour by diffusion through the pores of MOFs is conducive to a longer time of AcManNAz retention on the cell membrane.

ThS is a thioflavine dye that can selectively capture Aβ aggregates. To synthesize ThS with an alkyne terminus for the subsequent click reaction, ThS was first activated using SOCl_2_ to afford ThS–Cl.^37^ Then, propargyamine was added to react with sulfonyl chloride in ThS–Cl to obtain the alkyne-modified ThS (alk-ThS) (Fig. S21†).^38^ The strong –SO_2_–NH– vibrations (∼1162 cm^−1^) and the characteristic peaks of alkynyl (3292 cm^−1^ and 643 cm^−1^) in FT-IR demonstrated the successful synthesis of alk-ThS (Fig. S22†), and ^1^H NMR further demonstrated the successful preparation of alk-ThS (Fig. S23†).

Next, alk-ThS was conjugated with AcManNAz *via* the biorthogonal click reaction with catalytic Cu in Aβ plaques. Thus, the fixed alk-ThS on the microglial surface could serve as artificial Aβ receptors. As shown in Fig. S24,† CLSM imaging demonstrated the successful anchoring of ThS onto the microglial membrane owing to the emergence of the blue fluorescence of ThS.

**Fig. 3:**
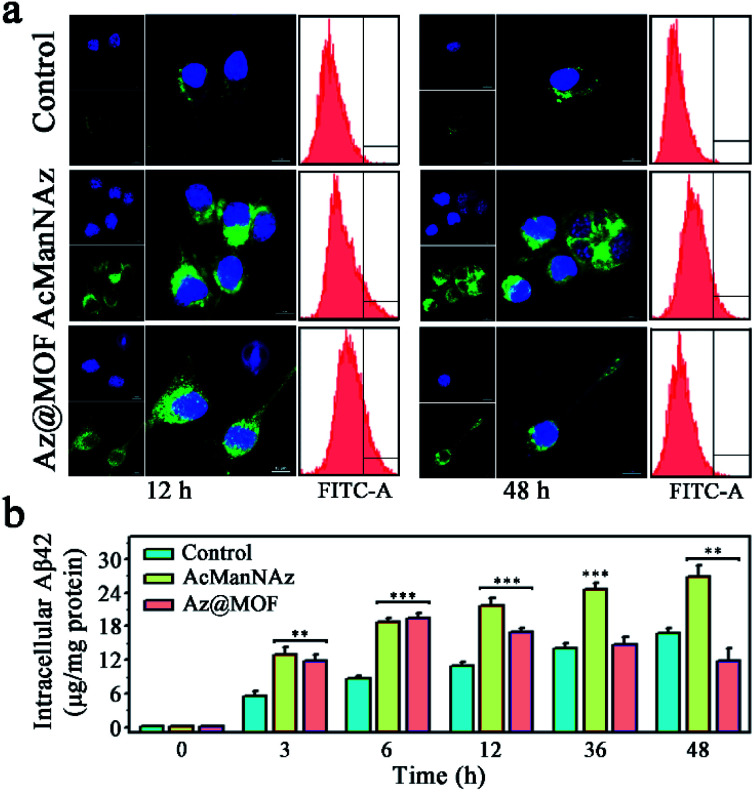
(a) Fluorescence images and flow cytometry assay of intracellular Aβ levels after 12 h or 48 h of incubation of Aβ with BV2 cells after pretreatment with PBS, AzManNAz, and Az@MOF (scale bar: 10 μm); (b) intracellular Aβ levels were quantified by ELISA and normalized to total protein at different time points after incubation with Aβ (*n* = 3; *P < 0.05, **P < 0.01, ***P < 0.001).

Microglial phagocytosis is the key mechanism for Aβ clearance. Microglial cell-surface receptors display a crucial role in phagocytosing Aβ. However, the expression of Aβ pattern recognition receptors of microglia such as SR, TLR, FPR, and RAGE would significantly decrease as AD progresses.^7^ Therefore, we guessed that the construction of artificial receptors on the microglial membrane might provide an alternative approach to capture Aβ to facilitate its phagocytosis. After the artificial Aβ receptors ThS were anchored onto the microglial membrane through metabolic glycoengineering and click reaction, the intracellular Aβ levels were assessed through CLSM images and flow cytometry assays. As shown in Fig. 3a, a significantly higher level of Aβ uptake was observed from the cells fed with AcManNAz and Az@MOF in the first 12 h after Aβ was added, and there was no obvious difference for AcManNAz and Az@MOF groups in the first 12 h except for a faster Aβ uptake observed in the group pretreated with AcManNAz than that with Az@MOF (Fig. S25†). The inspiring results validated our first surmise that artificially introducing the artificial Aβ receptors ThS onto the microglial surface helps facilitate Aβ phagocytosis. Normally, Aβ aggregates can be degraded *via* autophagy or ubiquitylation after phagocytosis by microglia. However, the level of intracellular Aβ elevated slowly and persistently with the increase of the time (12 h to 48 h) of co-incubation of Aβ and BV2 cells in the groups of AcManNAz (Fig. 3a and S25†). On the contrary, with further increase of time (12 h to 48 h), the level of intracellular Aβ gradually reduced in the microglia preincubated with Az@MOF (Fig. 3a and S25†). The same results were obtained through quantitative analysis of intracellular Aβ levels by enzyme-linked immunosorbent assay (ELISA) (Fig. 3b). These results collectively strongly indicated that engineering artificial receptors on the microglial membrane contributed to the phagocytosis of Aβ.

**Fig. 4:**
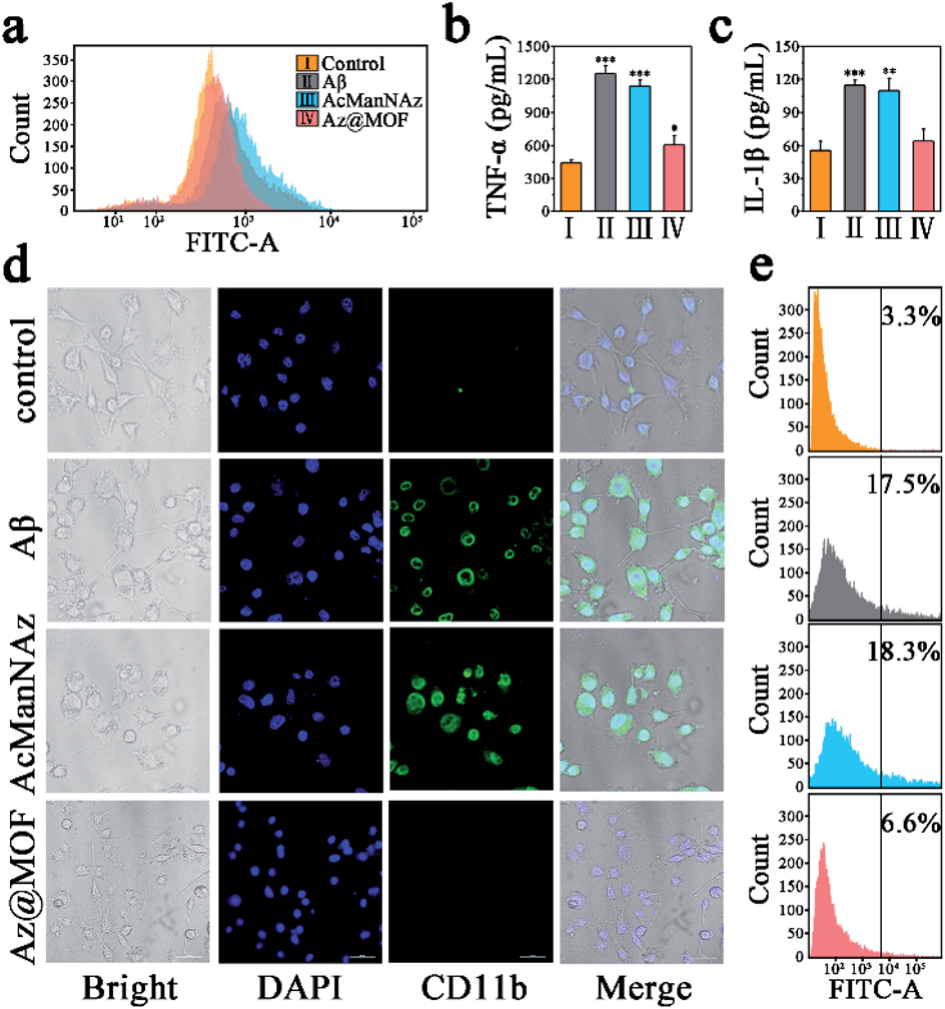
(a) Flow cytometry assay of intracellular ROS levels with DCFH-DA as the ROS probe after stimulation by Aβ aggregates; (b) quantitative analysis of TNF-α secretion and (c) IL-1β secretion of BV2 cells after stimulation by Aβ aggregates (*n* = 3; *P < 0.05, **P < 0.01, ***P < 0.001). The supernatants were collected for ELISA; (d) CLSM imaging CD11b positive BV2 cells by direct immunofluorescent staining (scale bar: 50 μm). CD11b is a microglial surface integrin expressed solely during activation. (e) Flow cytometry assay of CD11b positive BV2 cells.

A distinctly different consequence on intracellular Aβ levels was the slow and persistent increase of intracellular Aβ levels without Mn-MOFs as the carriers, while the arresting decrease of intracellular Aβ levels was observed with Mn-MOFs as the carriers. Previous research studies have already indicated that the antioxidative nanoparticles could scavenge multiple ROSs and drive microglial polarization from a pro-inflammatory phenotype to an anti-inflammatory phenotype, which is beneficial for Aβ clearance.^39,40^ In consideration of the only difference, that is, the presence or absence of Mn-MOFs with inherent SOD and CAT mimetic activity, we speculated that the antioxidant Mn-MOFs played an important role in lessening intracellular Aβ burden. Indeed, as shown in Fig. 4, Aβ deposition could act as the trigger to induce microglia to produce ROSs, secrete proinflammatory cytokines such as TNF-α and IL-1β and mediate microglial activation compared with the control group. Besides, microglia preincubated with AcManNAz could not only deteriorate inflammatory response but also slightly aggravate ROS production and proinflammatory cytokine secretion and CD11b expression during the process of Aβ phagocytosis and click reaction with Cu(i) as the catalyst. Nevertheless, the ROS production (Fig. 4a) and proinflammatory cytokine secretion (Fig. 4b and c) and CD11b expression (Fig. 4d and e) significantly reduced solely in the appearance of Mn-MOFs, which is attributed to their superior antioxidative ability. Besides, the amebiform BV2 cells recovered to their normal morphology with the pseudopodium. Collectively, these findings indicated that the enhanced Aβ clearance by microglia was the result of pretreating microglia with antioxidative Mn-MOFs. Furthermore, decreasing proinflammatory cytokines and avoiding the Aβ-mediated microglial activation by eradicating the over-produced ROS were conducive to normalize microglial dysfunction and improve the Aβ clearance.

Inspired by *in vitro* results, we evaluated the biocompatibility of Mn-MOF in a mouse model. Mn-MOF was stereotactically injected into the mouse brain. Then, we conduced terminal deoxynucleotidyltransferase-mediated nick-end labeling (TUNEL) staining of brain slices at two weeks post injection. The results showed no obvious cell apoptosis in the brain (Fig. S26†), and the hematoxylin and eosin (H&E) images further confirmed that there was no apparent harm to the brain and major organs (Fig. S27 and S28†). Furthermore, we conducted inductively coupled plasma mass spectrometry (ICP-MS) to verify the impact of Mn-MOF on the hemostases of key physiological ions including Cu, Zr and Mn (Table S2†). The cerebrospinal fluid (CSF) was collected respectively at 24 h and two weeks after Mn-MOF was stereotactically injected into the mouse brain. Compared to the control group (saline), a higher level of Mn and Zr can be detected in the CSF of Mn-MOF-treated mice 24 h after injection. However, the level of Mn and Zr kept almost the same as that of the control group two weeks after injection, which indicated that Mn-MOF was almost metabolized from the brain. Furthermore, the level of Cu showed slight reduction two weeks after injection, which may be due to high Cu affinity of TCPP.^41^ The results of ICP showed that Mn-MOF would not have a long-term impact on the hemostases of key physiological ions. So far, few studies have been carried out to reveal the fate of nanoparticles within the brain. The paravascular glymphatic pathway is a waste clearance system formed by astrocytes to promote efficient elimination of soluble proteins and metabolites from the brain through the exchange between cerebrospinal fluid and interstitial fluid, and the paravascular glymphatic pathway is the major route (about 80%) for brain elimination of the nanoparticles. Microglia-mediated transportation can facilitate their clearance through the paravascular route.^42^ Thus, we speculated that Mn-MOF would be eliminated from the brain mainly through microglia-mediated transportation and the paravascular glymphatic pathway. More detailed mechanisms on the clearance pathway of foreign nanomaterials from the brain are still unverified.

## Conclusion

In summary, we engineered microglial surfaces with Aβ aggregate-targeted ThS as artificial Aβ receptors *via* metabolic glycoengineering and click chemistry. The artificial Aβ receptors promoted microglia to phagocytose and clear up Aβ aggregates. What is more, Mn-MOFs, the carrier of AcManNAz, could mitigate oxidative stress, alleviate proinflammatory cytokine secretion and avoid microglial over-activation during the process of click reaction and Aβ phagocytosis by its admirable ability for scavenging ROSs. With the coordination between artificial Aβ receptors and antioxidative Mn-MOFs, the capability of microglia to phagocytose Aβ can speed up recovery without over-activated adverse effects. The present strategy by artificially introducing chemical Aβ-receptors onto the cell surface *via* metabolic glycoengineering and bioorthogonal chemistry is sufficient for accelerating microglia to eliminate Aβ, which also has valuable potential for other disease interventions associated with receptor starvation.

## Experimental

### Synthesis of Mn-MOFs

64 mg of 3, 180 mg of ZrOCl_2_·8H_2_O and 1.68 g of benzoic acid were dissolved in 60 mL of DMF in a 250 mL round bottom flask and the mixture was stirred at 90 °C for 4 h. After the reaction is complete, Mn-MOF nanoparticles were collected by centrifugation (12 000 rpm, 15 min) followed by washing with fresh DMF 3 times. The resulting Mn-MOF nanoparticles were suspended in DMF for further characterization and analysis.

### Synthesis of single crystalline

Mn-MOFs: 64 mg of Mn-TCPP, 180 mg of ZrOCl_2_·8H_2_O and 1.8 g of benzoic acid in 12 mL of DMF were ultrasonically dissolved in a Pyrex vial. The reaction mixture was heated in the 120 °C in an oven for 24 h. After being cooled to room temperature, dark purple crystals were harvested by filtration.

### Synthesis of Az@MOF

For the preparation of Az@MOF, 1 mL of AcManNAz (10 mg mL^−1^, 8 mg mL^−1^, 6 mg mL^−1^, 4 mg mL^−1^, and 2 mg mL^−1^ in alcohol) was mixed with 1 mL of Mn-MOFs (2 mg mL^−1^ in DMF) respectively. After sonication for 5–10 min, the resulting solution was stirred at 4 °C for 24 h. After that, the solid product was collected by centrifugation and then washed several times with water and alcohol (v/v 9 : 1). Then, the product was freezed-drying and stored at −20 °C for the next experiments. The loading amount of encapsulated AcManNAz in Mn-MOFs was determined by HPLC after complete lysis of Mn-MOFs by EDTA.

### Synthesis of ThS–Cl

ThS (1 g) was stirred in 20 mL of SOCl_2_ (containing 0.5 mL of DMF) at 70 °C for 24 h. The resulting yellow solution was then concentrated to dryness under reduced pressure. Ice water was added to the residue. After filtering, the brown-colored precipitate was washed with acetone three times. The remaining solid was dried at room temperature under vacuum, giving sulfuryl chloride-modified ThS.

### Synthesis of alk-ThS

The solid mixture of 2-propynylamine (0.18 g, 3.2 mmol) and 1.3 mL of triethylamine (9.7 mmol) was mixed in 15 mL CH_2_Cl_2_ in an ice bath. Then, 5 mL of ThS–Cl (1.84 g in CH_2_Cl_2_, 3.54 mmol) was added to the above solution and the mixed solution was stirred at room temperature. It took 24 hours for the reaction to complete and CH_2_Cl_2_ was added to dilute the product. The resulting organic layer was washed with saturated NaHCO_3_ solution and saturated NaCl solution and was dried over anhydrous Na_2_SO_4_ and evaporated to afford flavescent solid.

### Cell-surface glycan labeling

*N*-Azidoacetylgalactosamine (AcManNAz) and cyanine 5 alkyne were purchased from Sigma-Aldrich. BV2 cells were seeded in 6-well plates for flow cytometry experiments or 24-well plates for fluorescence microscopy analysis and cultured for 24 h to allow cell attachment. BV2 cells were cultured using the fresh MEM medium containing different concentrations of AcManNAz or Az@MOF for three days to enrich the azido groups in O-linked glycoproteins. The azido-labelled cells were washed three times with 1× PBS and then incubated with PBS containing 0.4 mM copper(ii), 2 mM sodium ascorbate, and 60 μM Cy5 alkyne in the dark at room temperature for 10 min, followed by three washes before fluorescence imaging or flow cytometry analysis. For the experiment of engineering artificial Aβ receptor ThS onto the microglial membrane, alk-ThS (50 μg mL^−1^) was used instead of Cy5 alkyne and 50 μM of AcManNAz or 60 μg mL^−1^ of Az@MOF was used.

### Aβ cellular phagocytosis

After the BV2 cells were labeled with ThS, followed by three washes with PBS, fresh 1% FBS containing MEM in the absence or presence of 12 μM of prepared Aβ42 aggregates was added. Then, the BV2 cells were incubated without or with Aβ42 aggregates at 37 °C for different times (3 h, 6 h, 12 h, 24 h, or 48 h) and followed by three washes with PBS to remove the dissociated Aβ. Then, antibodies against Aβ42 (Bioss) were applied to identify the Aβ cellular phagocytosis. The nucleus was stained blue with DAPI. For the experiment of quantification of the cellular level of Aβ, after washing with PBS, the cells were lysed in 1% SDS containing a protease inhibitor cocktail. The total protein content of cell lysates was analyzed *via* a bicinchoninic acid (BCA) protein assay (Beyotime), and the remaining intracellular Aβ42 levels were quantified with an ELISA kit (Bioss) and normalized to total protein in the lysates.

## Conflicts of interest

There are no conflicts to declare.

## Supplementary Material

SC-012-D0SC07067J-s001
