# Supplementary material for: Glycoengineering artificial receptors for microglia to phagocytose Aβ aggregates
Source: Chem Sci. 2021 Feb 23;12(13):4963–9. doi: 10.1039/d0sc07067j (PMC8179537; doi:10.1039/d0sc07067j)
Supplement: SC-012-D0SC07067J-s001 [file SC-012-D0SC07067J-s001.pdf]

Electronic Supplementary Material (ESI) for Chemical Science.
This journal is © The Royal Society of Chemistry 2021

Supporting Information

Glycoengineering Artificial Receptors for Microglia to Phagocytose Aβ aggregates

Dongqin Yu,[a,b] *Chun Liu,*[a,b] *Haochen Zhang,*[a,b] *Jinsong Ren,*[a,b] *and Xiaogang Qu**[a,b]

Chemicals: All reagents were of analytical reagent grade and used without any further purification. Organic solvents were purchased from Beijing Chemical Works. Other chemicals were purchased from TCI, Sigma-Aldrich, or Aladdin. Aβ42 was purchased from ChinaPeptides Co., Ltd. mOC78. All other chemicals were obtained from Sigma Aldrich and used as supplied.

Measurements: The UV-Vis absorption spectra were recorded using a JASCO V-550 UV/Visible spectrophotometer (JASCO International Co., LTD., Tokyo, Japan). Fluorescence spectroscopy measurements were conducted on JASCO FP-6500 spectrofluorometer. Scanning electron microscopic (SEM) images were recorded using a Hitachi S-4800 Instrument (Japan). Transmission electron microscopic (TEM) images of cells were captured with a FEI TECNAI G2 20 highresolution transmission electron microscope operating at 200 kV. The crystalline structures of the as prepared samples were evaluated by X-ray diffraction (XRD) analysis on a Rigaku-Dmax 2500 diffractometer by using CuKα radiation. The operation voltage and current were kept at 40 kV and 40 mA. N_2 sorption isotherms at 77 K were measured by using a Micrometritics ASAP 2420 system with high-purity grade (99.999%) of gases. 1H NMR spectrum was recorded on a Bruker-600 MHz NMR instrument. X-ray photoelectron Spectroscopy (XPS) spectra were analyzed by Thermo Fisher Scientific ESCALAB 250Xi Spectrometer Electron Spectroscopy (America). The flow cytometry data was obtained by BD LSRFortessa™ Cell Analyzer. Fourier transform infrared spectroscopy (FTIR) spectra were recorded on a BRUKER Vertex 70 FTIR

spectrometer. Fluorescence microscopy images were obtained using a JASCO FP-6500 spectrofluorometer with the slit width for the excitation and emission of 3 nm. Electrospray ionization (ESI) mass spectra (MS) were acquired using the Quattro Premier XE mass spectrometer (Waters, USA).

Synthesis of 5,10,15,20-Tetrakis(4-methoxycarbonylphenyl)porphyrin (TPPCOOMe, 1): 100 mL of propionic acid in a 500 mL three necked flask were added 3.0 g of pyrrole and 6.9 g of methyl *p*-formylbenzoate, and the solution was refluxed at 140 °C for 12 h in darkness. After the reaction mixture was cooled to room temperature, crystals were collected by suction-filtration to afford purple crystals.

Synthesis of 5,10,15,20-Tetrakis(4-methoxycarbonylphenyl)porphyrin-Mn (Mn-TPPCOOMe, 2): 0.854 g of 1 and 2.5 g of $MnCl_2·4H_2O$ in 100 mL of DMF was refluxed for 6 h in darkness. After the mixture was cooled to room temperature, 150 mL of H_2O was added. The resultant precipitate was filtered and washed with 50 mL of H_2O for two times. The obtained solid was dissolved in $CHCl_3$, followed by washing three times with water. The organic layer was dried over anhydrous magnesium sulfate and evaporated to afford quantitative dark green crystals.

Synthesis of [5,10,15,20-Tetrakis(4-carboxyphenyl)porphyrinato]-Mn (Mn-TCPP, 3): 0.75 g of 2 was stirred in 25 mL of THF and 25 mL of MeOH mixed solvent. Then 25 mL of KOH (46.95 mmol) solution was introduced. This mixture was refluxed for 12 h in darkness. After cooling down to room temperature, THF and MeOH were evaporated. Additional water was added to the resulting water phase and the mixture was heated until the solid was fully dissolved, then the homogeneous solution was acidified with 1M HCl until no further precipitate was detected. The dark green solid was collected by filtration, washed with water and dried in vacuum.

SOD Mimetic Enzyme Activity: The SOD activities were assayed by measuring inhibition of the photoreduction of nitro blue tetrazolium (NBT). The Mn-MOFs with various concentrations was mixed with riboflavin (20 μM), methionine (0.013 M),

and NBT (75 μM). The mixtures were illuminated by a lamp with a constant light intensity for 10 min at 25℃. After illumination, the absorbance spectrum was recorded. Identical tubes with the reaction mixture were kept in the dark and served as blanks. For comparison the SOD activity of Mn-MOFs and Mn-TCPP, a SOD assay kit (Beyotime, Shanghai, China) was used and the absorbance at 540 nm was measured with an automatic plate reader.

CAT Mimetic Enzyme Activity: The CAT activities were assayed by measuring inhibition of the generation of 2-hydroxyterephthalic acid. The Mn-MOFs and Mn-TCPP with various concentrations was mixed with terephthalic acid (TA) (0.5 mM), H_2O_2 (10 mM). The mixture was incubated at 37 ℃ for 24 h. TA is a non-fluorescent compound which can capture OH• to produce the highly fluorescent 2-hydroxyterephthalic acid with an emission peak at 425 nm upon excitation wavelength of 320 nm. For comparison the CAT activity of Mn-MOFs and Mn-TCPP, the same experimental procedure was conducted the fluorescence intensity at 425 nm was measured with an automatic plate reader.

Preparation of Aβ42 Aggregates: Aβ42 was provided ChinaPeptides Co., Ltd. mOC78. The sample was prepared as previously described. Briefly, the powdered Aβ42 was dissolved in 1,1,1,3,3,3-hexafluoro-2-propanol solution at a concentration of 1 mg mL^{-1} (240 μM). The solution was shaken in a 4 °C freezer for 4 h for sufficient dissolution and then stored at -20 ℃ as a stock solution. Before use, the solvent 1,1,1,3,3,3-hexafluoro-2-propanol was removed by evaporation with a gentle stream of nitrogen. The peptide was dissolved in Milli-Q water. Aggregates formation was carried out at 37 ℃ incubator with or without Cu. The presence of aggregates in these preparations has been previously confirmed and characterized with CD spectrum.

Cell Culture: BV2 cells, obtained from American Type Culture Collection, were grown on plates in MEM media containing 10% fetal bovine serum, 1% serum L-glutamate and 1% streptomycin in a humidified atmosphere of 5% CO2 and 95%

air. The media were changed every three days, and the cells were passaged by trypsinization before confluence.

Cytotoxicity Assays: Cell viability was measured by 3-(4,5-dimethylthiazol-2-yl)-2,5-diphenyltetrazoliumbromide (MTT) assays. BV2 cells were seeded at a density of 5000 cells/well on 96-well plates for 24 h. Cells were incubated for 24 h and were treated with the Mn-MOFs with different concentration and then MTT assays were carried out. To determine toxicity, 10 μL of MTT solution was added to each well and the cells were incubated for 4 h. After adding 100 μL DMSO, the absorbance of formazan was read at 490 nm on a SpectraMax M5 microplate reader. Three replicates were done for each treatment group.

Cellular Uptake of the Nanoparticles: RhB@MOF was coincubated with preseeded BV2 cells in 24-well plates for fluorescence microscopy analysis or 6-well plates for flow cytometry experiments for different time. After 24 h incubation, old media were removed and cells were washed three times with PBS. The confocal laser scanning microscopy (CLSM) was employed to image the cellular distribution of RhB to verify the cellular uptake of the Mn-MOFs. For the cells in 6-well plates, after different time of incubation, the cells were washed with PBS twice, and the fluorescence intensity of cells was monitored by flow cytometry (BD LSRFortessa™ Cell Analyzer).

Immunofluorescent Staining: After incubation, BV2 cells were fixed in 10% neutral buffered formalin for 30 min and rinsed 3 times in PBS for 5 min. Cells were blocked in 0.1% triton-X and 10% BSA in PBS for 1 h and then incubated in anti-Aβ42 antibody 1:500 in PBS overnight at 4 °C. Cells were rinsed 3 times in PBS for 5 min before incubation with FITC-conjugated secondary antibodies (1:500, FITC-conjugated goat anti-rat, Bioss Antibodies) for 2 h at room temperature. Immunofluorescence images were acquired with a Nikon Confocal Microscope (A1R MP).

Measurement of Inflammatory Cytokines Level: After the BV2 cells were labeled with ThS, BV-2 cells were stimulated with different Aβ aggregates for overnight. The

supernatants were collected and stored at -20 °C until assays for TNF-α and IL-1β were performed. TNF-α and IL-1β levels were detected by mouse TNF-α ELISA kits and IL-1β ELISA kits according to the procedures provided by the manufacturers.

Measurement of Level of CD11b: After treatment with Aβ42 aggregates, BV2 cells were blocked in 0.1% triton-X and 10% BSA in PBS for 1 h and then incubated in primary antibody CD11b 1:500 (Rat polyclonal) in PBS overnight at 4 °C. Cells were rinsed 3 times in PBS for 5 min before incubation in secondary goat anti-rat (495 nm) at a 1:1,000 dilution of for 1 h. Glass coverslips were rinsed 3 times in PBS prior to adhesion to microscope slides. Immunofluorescence images were acquired with a Nikon Confocal Microscope (A1R MP) and flow cytometry results were obtained by BD LSRFortessa™ Cell Analyzer.

Handling of mice: All animal experiments were performed according to the NIH guidelines for the care and use of laboratory animals (NIH Publication No. 85-23 Rev. 1985) and approved by the Jilin University Animal Care and Use Committee. Eight- to 12-week-old Kunming mice (sex-randomized) were used as test animals. The data was expressed as mean ± standard deviation (SD) and performed in at least 3 specimens (n=3). W Kunming mice stereotactically injected bilaterally with 5 μL of Mn-MOF (5mg/mL) at a rate of 1 μL/3 min (RWD Life Science Co. Ltd Shenzhen). The injection coordinates, relative to Bregma, were anteroposterior, −1.70 mm; mediolateral, 1.50 mm; and dorsoventral, −1.50 mm. After anesthesia and exposure of the cisterna magna, CSF was obtained using a glass-pulled micropipette at 24 h or two weeks post-injection, ensuring that the CSF was not contaminated with blood. About

10 ml of the CSF was obtained. The CSF was immediately diluted 10-fold in 1% CHAPS (3-[(3-cholamidopropyl)dimethylammonio]-1-propanesulfonate) in PBS with protease inhibitors (Roche Diagnostics). The Cu, Mn, and Zr content of the samples was measured by ICP-MS (Varian 720-ES). At the same time, the whole brains and major organs including heart, liver, spleen, lung and kidney were harvested and fixed in 10% formalin, embedded in paraffin, and sectioned at two weeks post-injection. Terminal deoxynucleotidyltransferase-mediated nick-end labeling (TUNEL) staining of brain slices and hematoxylin and eosin (H/E) of brain and major organs was conducted.

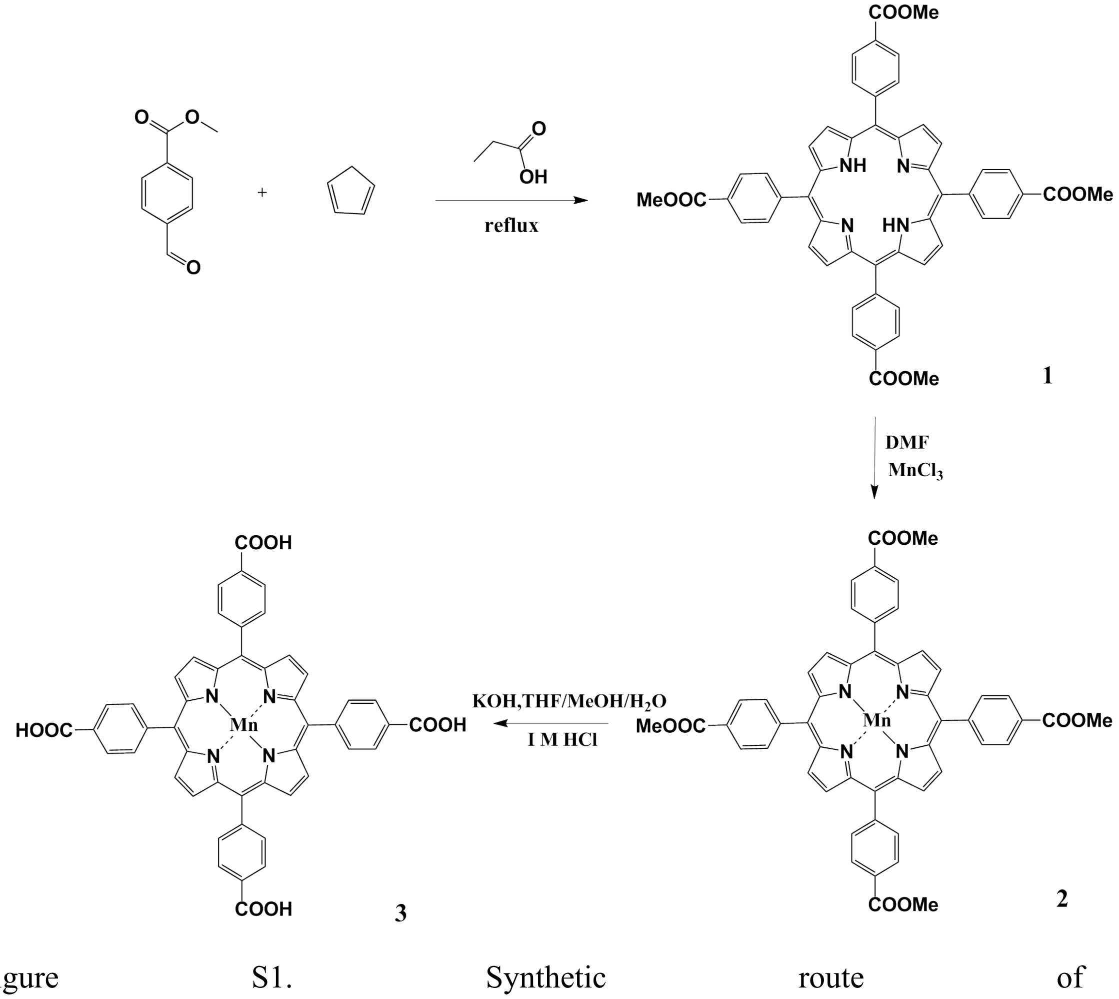

Figure S1. Synthetic route of

[5,10,15,20-Tetrakis(4-carboxyphenyl)porphyrinato]-Mn (Mn-TCPP, 3).

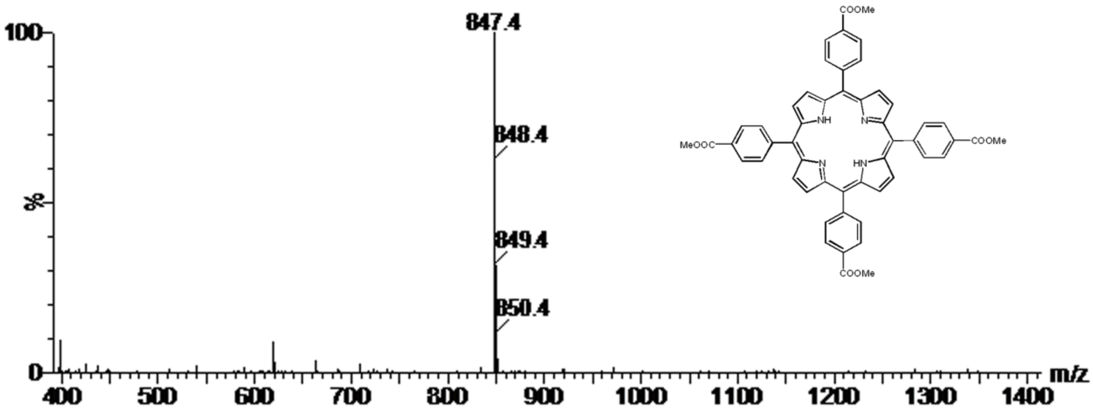

Figure S2. Mass spectrum (MS) of 5,10,15,20-Tetrakis(4-methoxycarbonylphenyl)porphyrin (TPPCOOMe, 1).

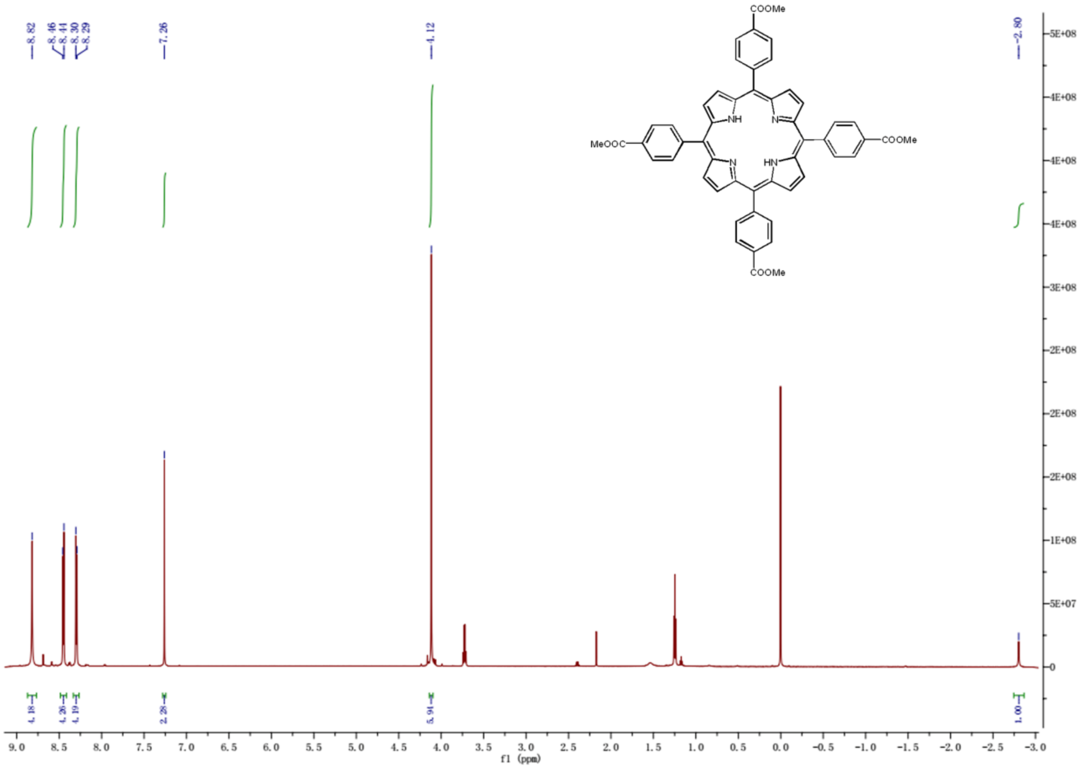

Figure S3. 1H NMR of 5,10,15,20-Tetrakis(4-methoxycarbonylphenyl)porphyrin (TPPCOOMe, 1).

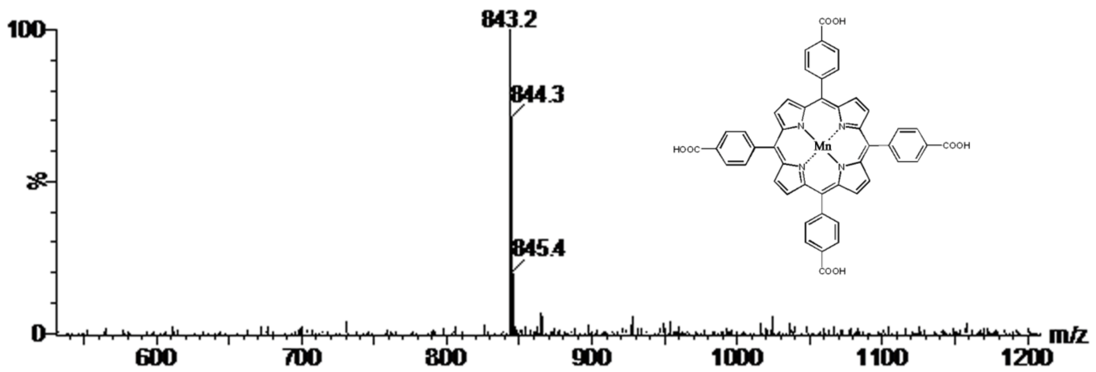

Figure S4. Mass spectrum (MS) of [5,10,15,20-Tetrakis(4-carboxyphenyl)porphyrinato]-Mn (Mn-TCPP, 3).

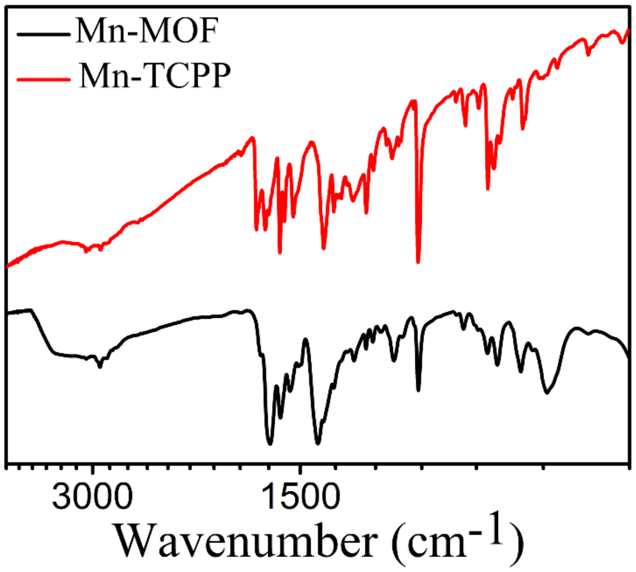

Figure S5. FT-IR spectra for Mn-TCPP and Mn-MOFs.

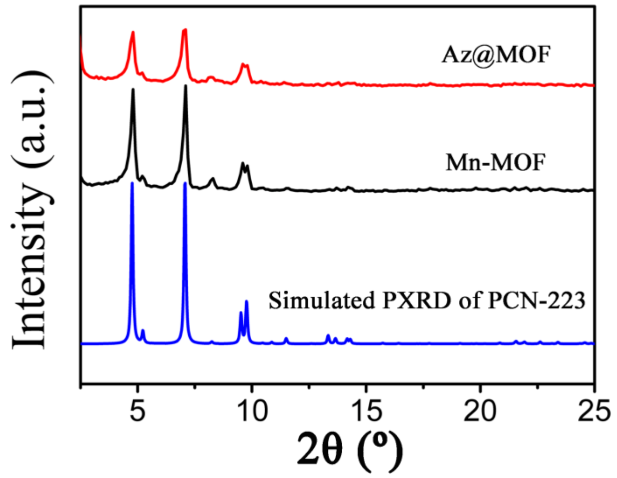

Figure S6. PXRD of Mn-MOF and Az@MOF.

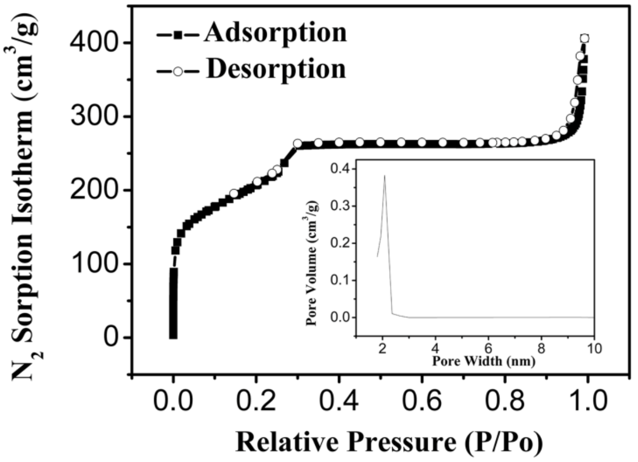

Figure S7. N_2 adsorption isotherms of the Mn-MOFs. Inset: Pore size distribution of the Mn-MOFs calculated by the JHB model.

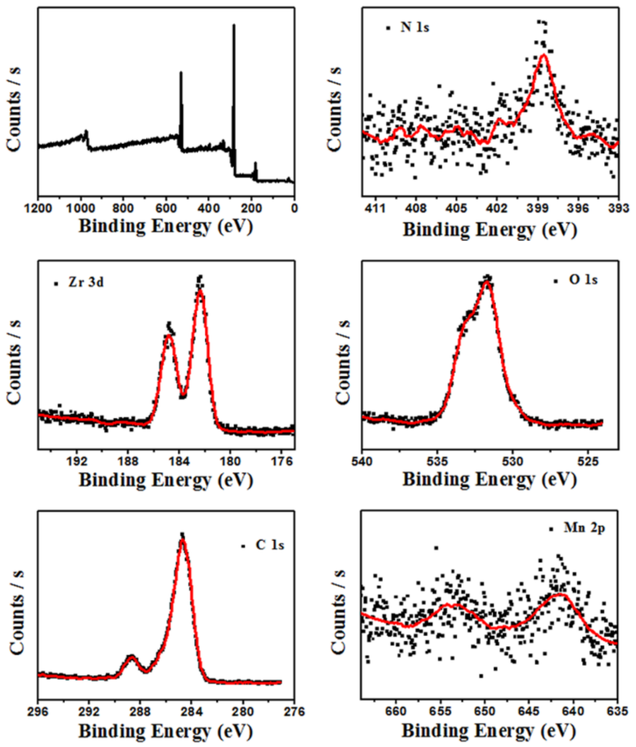

Figure S8. XPS spectrum of Mn-MOFs.

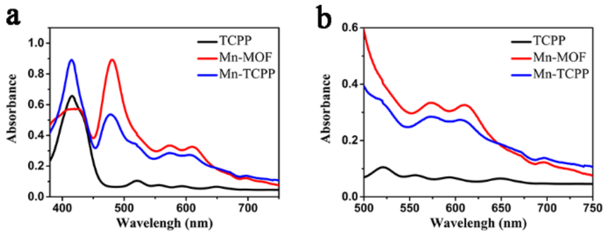

Figure S9. a) UV−vis spectra of TCPP, Mn-TCPP, and the Mn-MOFs; b) Amplification of the UV−vis spectra of TCPP, Mn-TCPP, and the Mn-MOFs at 500−750 nm.

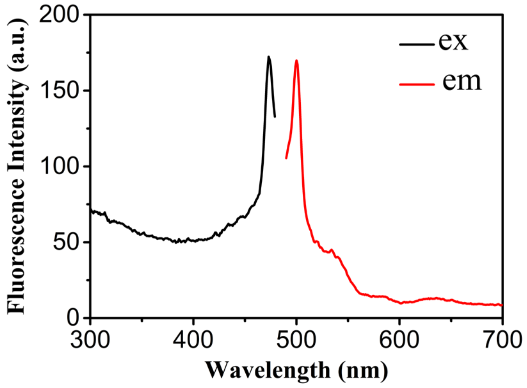

Figure S10. Fluorescence excitation and emission spectra of Mn-MOF.

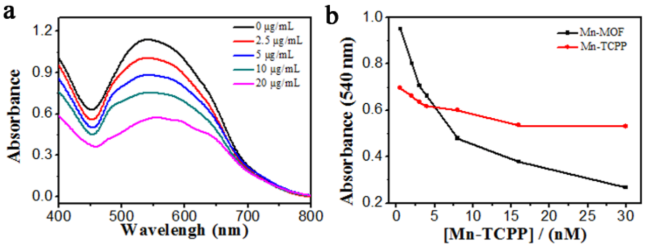

Figure S11. a) The SOD activity of Mn-MOFs with different concentration measured by the inhibition of NBT oxidation; b) The comparison of SOD activity of Mn-MOFs and Mn-TCPP with same concentration of Mn-TCPP.

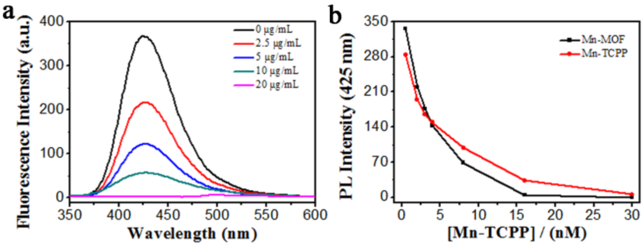

Figure S12. a) The CAT activity of Mn-MOFs with different concentration measured by inhibition of TA oxidation; b) The comparison of CAT activity of Mn-MOFs and Mn-TCPP with same concentration of Mn-TCPP.

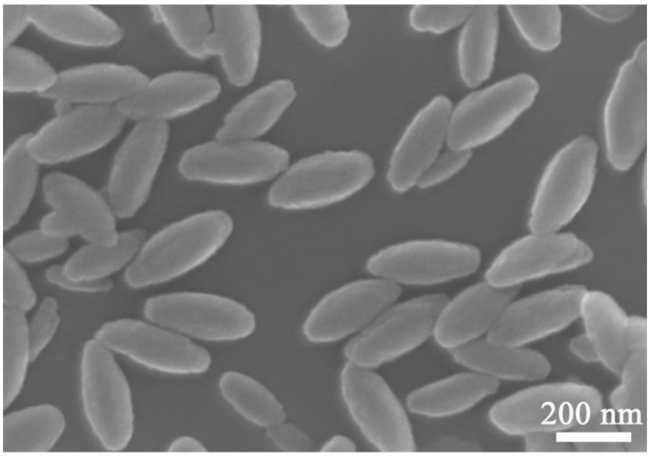

Figure S13. SEM image of Az@MOF.

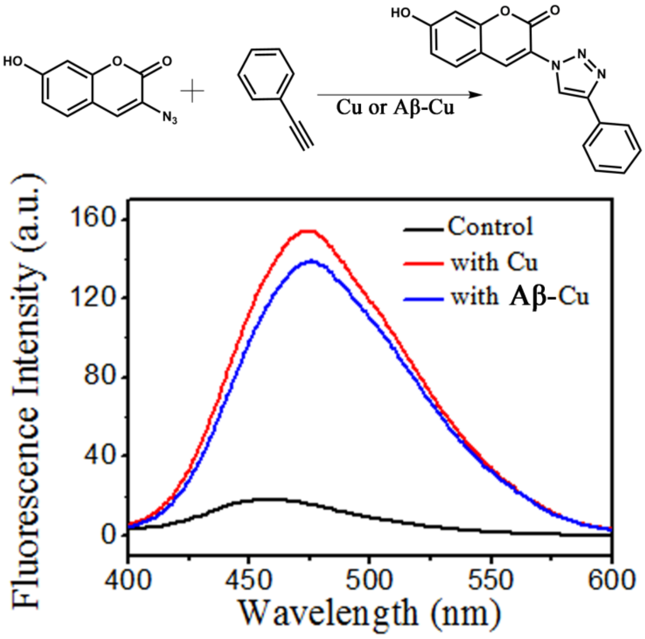

Figure S14. The changes of fluorescence spectra without catalyst or with Cu or Aβ-Cu aggregates as catalysts.

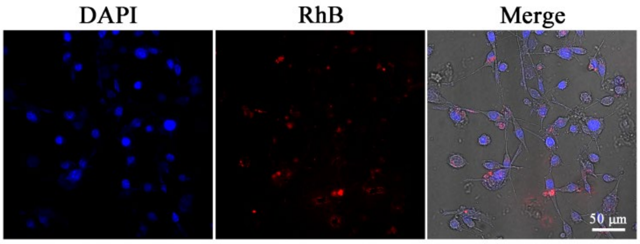

Figure S15. Internalization of Mn-MOFs by microglia with RhB as a fluorescence probe.

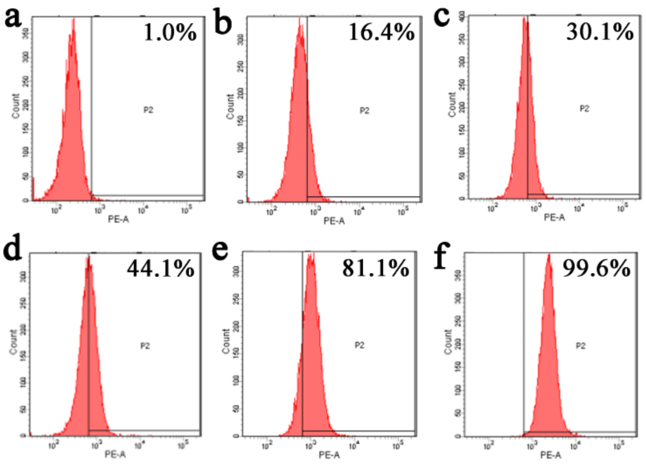

Figure S16. Flow cytometry assay of release of Mn-MOFs by microglia with RhB as a model drug and fluorescence probe after co-incubation RhB@MOFs with microglia with different time (a. control; b. 1 h; c. 3 h; d. 12 h; e. 24 h; f. 48 h after RhB@MOFs was added).

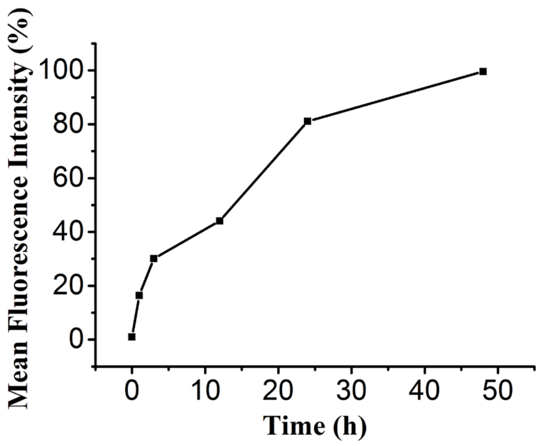

Figure S17. Quantitative analysis of flow cytometry of release of RhB after RhB@MOFs were endocytosed by microglia.

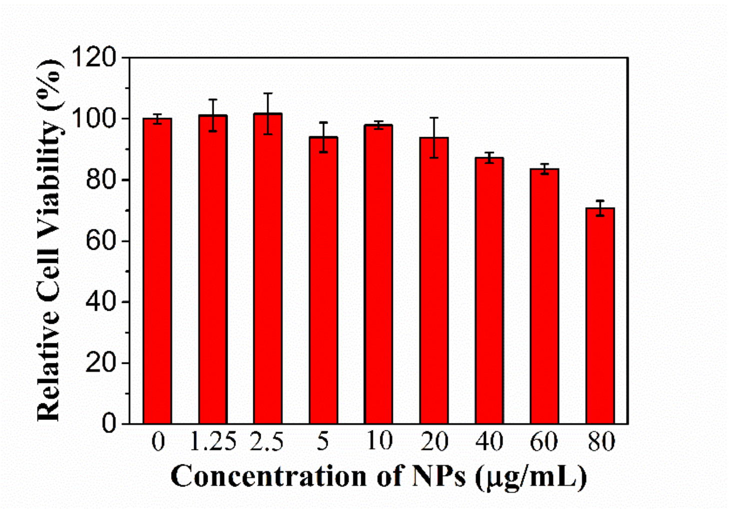

Figure S18. MTT assay of microglia treated with different concentration of Mn-MOFs.

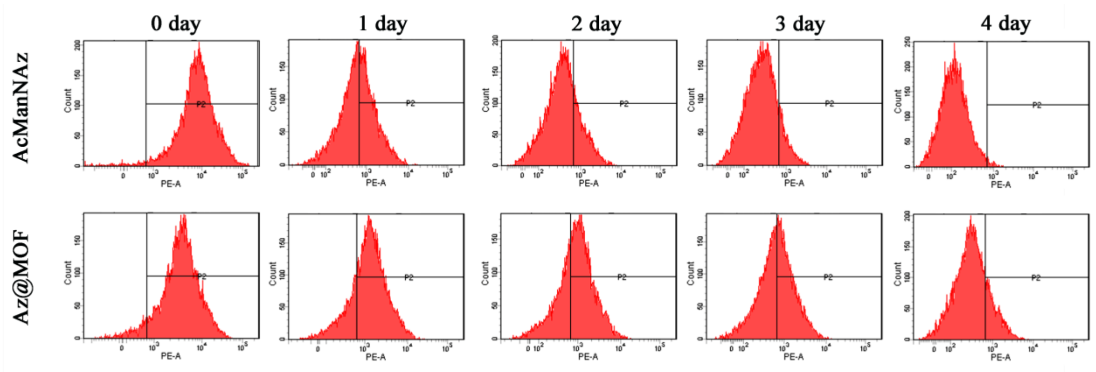

Figure S19. Flow cytometry assay of amount of the azido groups on the microglial membrane with different time.

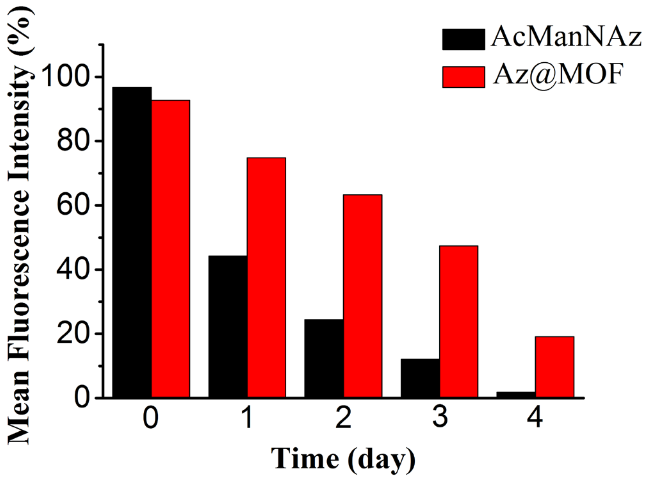

Figure S20. Quantitative analysis of flow cytometry assay of amount of the azido groups on the microglial membrane with different time.

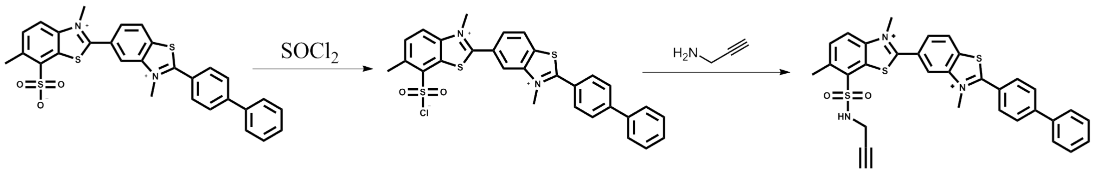

Figure S21. Synthetic route of Alk-ThS.

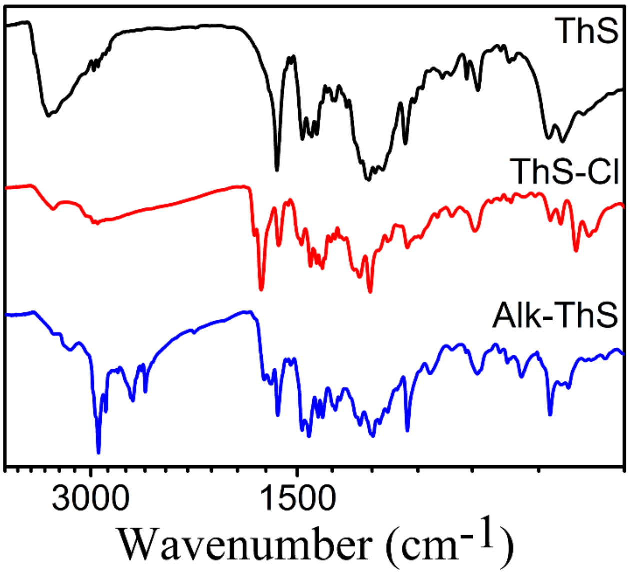

Figure S22. IR spectra of ThS, ThS-Cl and alk-ThS.

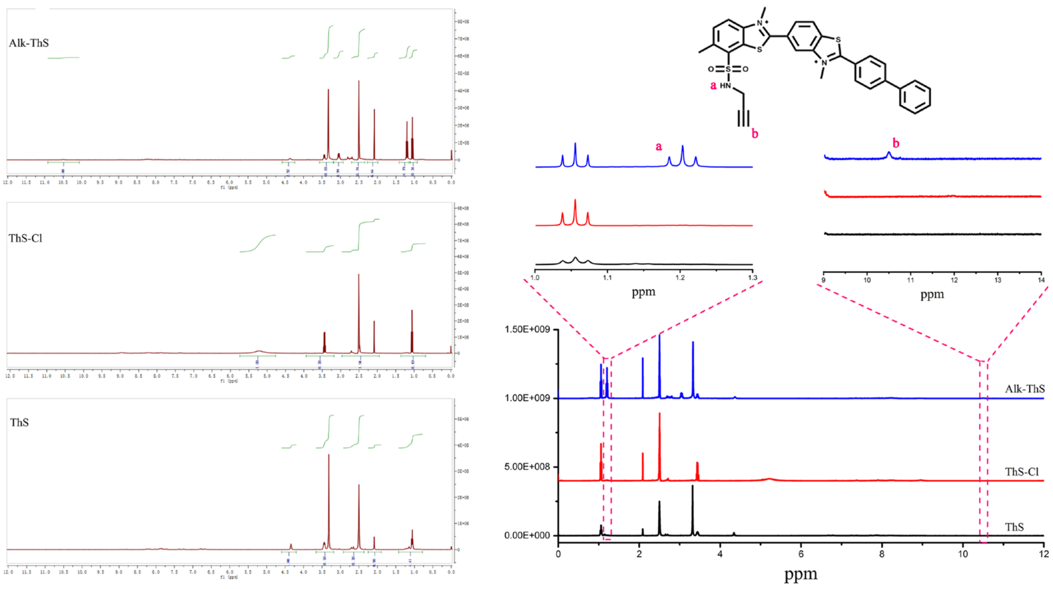

Figure S23. ^{1}H NMR of ThS, ThS-Cl and alk-ThS.

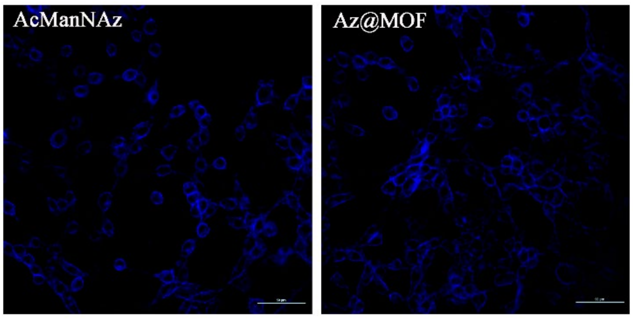

Figure S24. Imaging ThS on the microglial membrane with AcManNAz or Az@MOF (Scale bar: 50 μm).

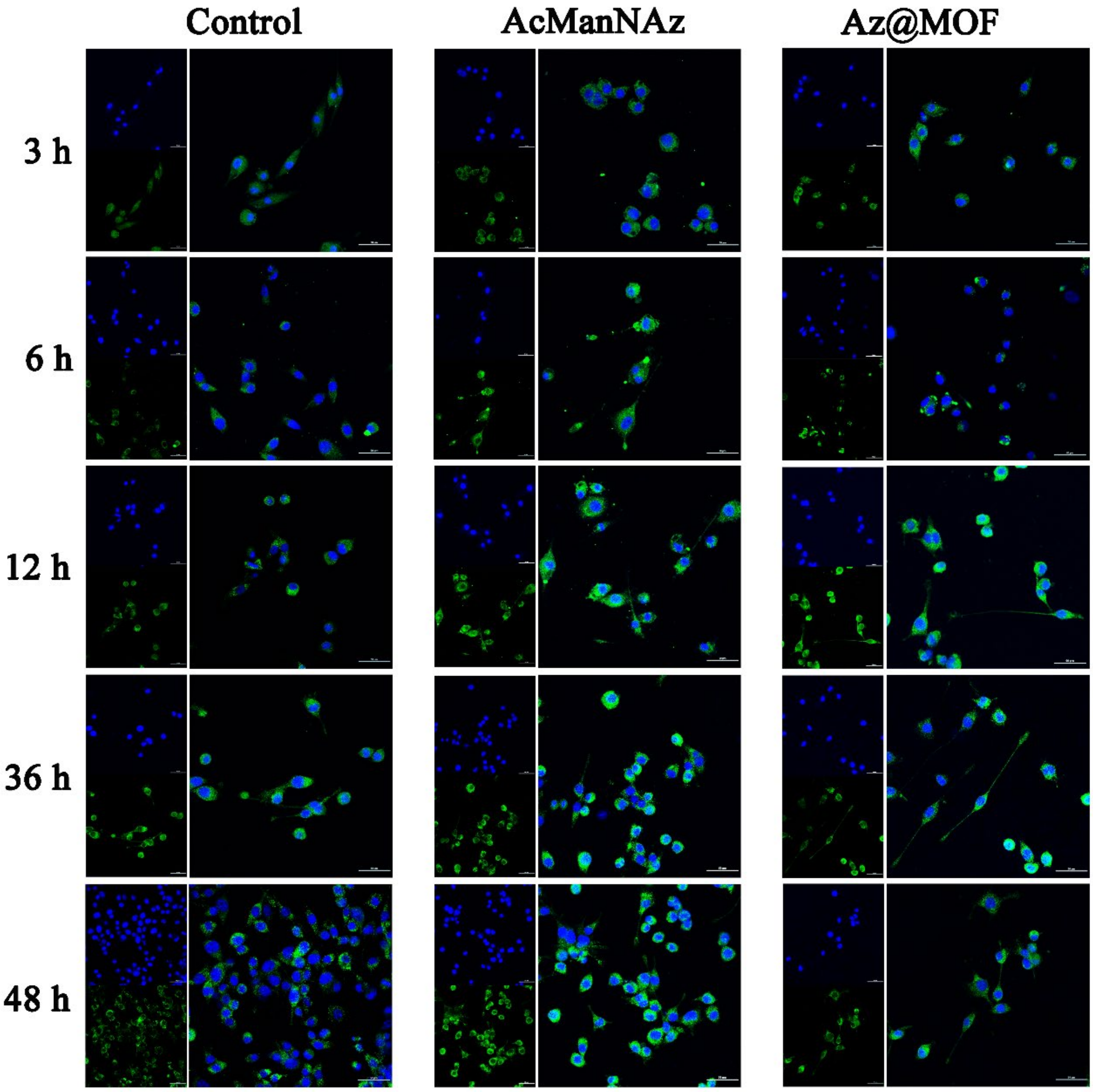

Figure S25. Fluorescence images of intracellular Aβ levels after different time of incubation of Aβ with BV-2 cells after pretreatment with PBS, AcManNAz, and

Az@MOF and click reaction (Scale bar: 50 µm).

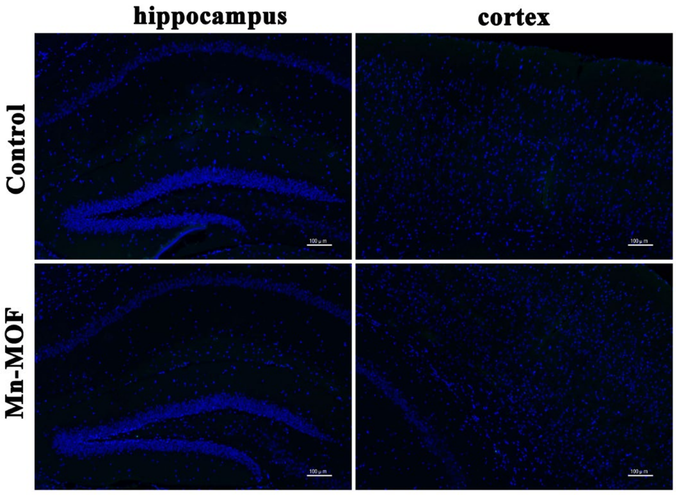

Figure S26. TUNEL staining of brain slices after two weeks of Mn-MOF treatment. Green: TUNEL-stained apoptosis cells. Blue: DAPI-labeled nucleus (scale bar = 100µm) (n = 3 mice per group).

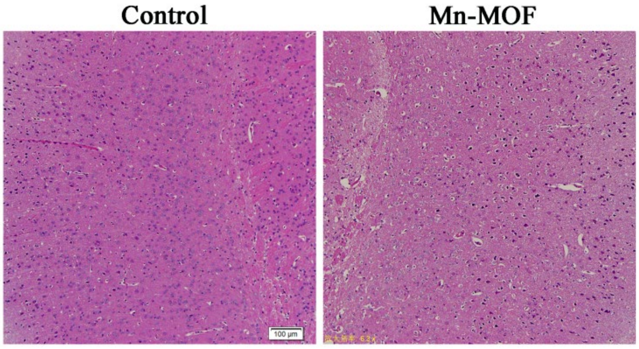

Figure S27. H&E staining of brain after two weeks of Mn-MOF treatment (scale bar = 100 µm) (n = 3 mice per group).

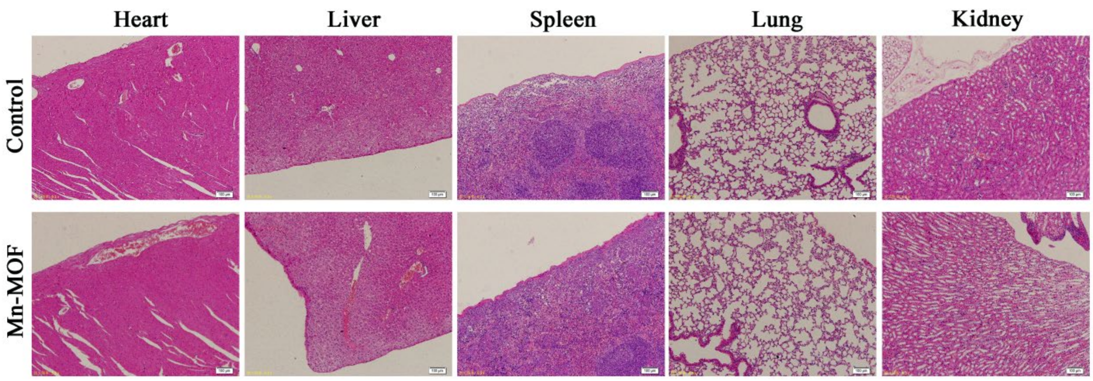

Figure S28. H&E staining of major organs after two weeks of Mn-MOF treatment (scale bar =100 μm) (n = 3 mice per group).

Table S1. The amount of encapsulated AcManNAz mearsured by HPLC.

sample	1	2	3	4	5
AcManNAz:Mn-MOFs (μmol/mg)	11.6279	9.3023	6.9767	4.6512	2.3256
Amount of encapsulated AcManNAz (μmol/mg)	0.85	0.83	0.67	0.59	0.33

Table S2. The concentration of Zr, Mn, and Cu in the CSF at 24 h and 5 weeks post-injection (n = 3 mice per group).

	24 h after injection			2 weeks after injection		
	Zr (μg/L)	Cu (μg/dL)	Mn (μg/dL)	Zr (μg/L)	Cu (μg/dL)	Mn (μg/dL)
Saline	N/D	15.25±4.15	2.04±0.15	N/D	15.53±4.44	1.94±0.14
Mn-MOF	1.27±0.19	15.46±2.66	2.22±0.23	N/D	13.72±1.89	2.07±0.21
